# Multi-Omics Integration to Investigate the Effects of Variety and Origin on Volatile and Non-Volatile Metabolites in Melons

**DOI:** 10.3390/foods15101746

**Published:** 2026-05-15

**Authors:** Junzhe Hua, Kemin Mao, Wenlong Yu, Zongyang Li, Runhan Wen, Lingyu Li, Liyan Song, Yaxin Sang, Xianghong Wang

**Affiliations:** 1 College of Food Science and Technology, Hebei Agricultural University, No. 2596 Lekai South Street, Baoding 071000, China; 15719016123@163.com (J.H.); keminmao@outlook.com (K.M.); yuwenlong0810@163.com (W.Y.); 13931226615@163.com (Z.L.); 13184750826@163.com (R.W.); lly11022024@163.com (L.L.); sangyx1418@163.com (Y.S.); 2 Laboratory of Dietary Component Interactions and Precision Nutrition, Baoding 071000, China; 3 Agriculture and Rural Affairs Bureau of Qing County, Cangzhou 062650, China; 15735861061@163.com

**Keywords:** saline–alkali soil, *Cucumis melo* L., flavor differences, metabolomics, integrative multi-omic

## Abstract

This study investigated the effects of different cultivation environments on melon quality development and the underlying metabolic regulatory mechanisms. Using ‘Yangjiaocui’ and ‘Boyang 9’ melons, we systematically compared their physicochemical properties, nutritional components, volatile compounds, and metabolites under saline–alkali versus normal conditions, employing an integrated multi-omics analytical model. The results showed that saline–alkali cultivation significantly increased several nutritional components (e.g., soluble solids, vitamin C, flavonoids, and polyphenols) compared to normal conditions. Gas chromatography–ion mobility spectrometry (GC-IMS) detected 36 volatiles, predominantly esters and ketones, with 13 key markers such as isovaleric acid isovaleryl ester and ethyl butyrate, effectively discriminating cultivars and growth origins. Liquid chromatography–mass spectrometry (LC-MS) detected 702 metabolites, chiefly organic acids and lipids. KEGG pathway enrichment analysis revealed that flavonoid biosynthesis was the most significantly enriched pathway (enrichment factor ~1, extreme significance), with coordinated regulation of tyrosine and phenylalanine metabolism redirecting metabolic flux toward defensive secondary metabolites. In conclusion, our results suggest that saline–alkali cultivation may contribute to improved nutritional profiles, and multi-omics analysis effectively differentiates melon varieties and origins. This study provides a theoretical basis for understanding the quality, flavor, and metabolite profiles of melon under saline–alkali stress, employing a multi-omics approach.

## 1. Introduction

Melon (*Cucumis melo* L.), an annual herbaceous species within the Cucurbitaceae family, is indigenous to Africa and extensively cultivated throughout Asia. As a globally significant cash crop, it is characterized by a short growth cycle, high benefit-to-cost ratio, and substantial nutritional value, contributing to its considerable consumer popularity [1]. The fruit is distinguished by its crisp texture and pronounced aroma, qualities that endow it with notable economic value and market potential. In Hebei Province, melon cultivation covers an area of 1.546 million mu (approximately 103,000 hectares), yielding an output value of 14.25 billion CNY. Both the cultivation area and total production rank among the highest in the nation, making melon production a crucial income source for local farmers. However, the extensive diversity among melon varieties leads to considerable variation in quality, flavor, and environmental adaptability. This heterogeneity underscores the necessity for scientific evaluation and selection of high-quality cultivars suited to specific agro-ecological conditions, which has emerged as a key research focus. Among the widely cultivated varieties, ‘Yangjiaocui’ and ‘Boyang 9’ represent two mainstream types. ‘Yangjiaocui’ is characterized by an oblong fruit shape, relatively thick rind, traditional flavor, and distinctive appearance [2]. In contrast, ‘Boyang 9’ exhibits smooth peel, thick flesh, high sugar content, and a crisp, sweet taste.

Although previous studies have confirmed significant differences in nutritional components (such as minerals and vitamin C) among different melon varieties [3], the current evidence is primarily derived from conventional cultivation. As a typical abiotic stress environment, saline–alkali soil induces specific salt stress and ion toxicity that directly interfere with physiological processes and the biosynthesis of secondary metabolites. These effects are likely to alter the nutritional quality and bioactive composition of melon fruits. Nevertheless, systematic comparisons of varietal adaptation mechanisms and their associated effects on fruit nutritional quality under saline–alkali conditions remain limited. This gap constrains the targeted and effective use of saline–alkali land for quality melon production. Thus, future studies should prioritize evaluating varietal adaptability under saline–alkali stress, clarifying the response of nutritional traits and developing strategies for efficient resource use. Such work will provide theoretical insights into melon salt tolerance physiology and support the sustainable development of specialized melon production in saline–alkali regions.

In recent years, volatile compounds in fruits have been extensively investigated using various analytical techniques, particularly headspace solid-phase microextraction coupled with gas chromatography-mass spectrometry (HS-SPME-GC-MS). Previous studies have characterized volatile profiles in fruits such as peaches [4] and mangoes [5], establishing relationships between specific aroma compounds and flavor quality. For example, Valverde et al. [6] investigated the relationship between aroma components and sensory attributes in melons cultivated in La Mancha, Spain, over three consecutive growing seasons; significant correlations were identified between volatile compounds and specific sensory descriptors such as fruity and floral notes. While existing research has advanced the understanding of melon volatile composition and characteristics, most studies continue to rely exclusively on GC-MS methodologies. Although GC-MS is sensitive, it often requires extensive sample preparation and may fail to detect certain classes of volatiles owing to extraction or ionization biases, potentially leading to an incomplete representation of the volatile profile. Moreover, the flavor profiles of fruits cultivated under saline–alkaline conditions remain largely unexplored. In contrast to GC-MS, GC-IMS requires minimal sample preparation, enables rapid analysis, and is particularly suitable for detecting volatile organic compounds. Thus, applying GC-IMS to characterize the composition and distinctive features of volatile compounds in melons grown under saline–alkaline conditions can yield complementary insights that are not accessible by conventional GC-MS alone.

Non-targeted metabolomics has gained extensive application in fruit research in recent years. It has demonstrated particular significance in quality evaluation, flavor profiling, and metabolite analysis [7]. By enabling comprehensive detection and systematic analysis of metabolites, this approach not only reveals the composition, variation, and dynamic patterns of metabolic profiles but also provides crucial theoretical and technical support for quality assessment, flavor characterization, and breeding of superior fruit varieties. However, the application of non-targeted metabolomics in melon research remains relatively limited [8]. Existing studies have yet to establish a systematic framework, warranting further development in areas such as metabolite database construction, key metabolic pathway elucidation, and understanding the regulatory mechanisms through which environmental and genetic factors shape the melon metabolome.

This study systematically investigated the physicochemical properties (color, texture, basic nutrients, and mineral elements) of two melon cultivars (‘Yangjiaocui’ and ‘Boyang 9’) grown in saline–alkali versus non-saline–alkali soils. Volatile flavor profiles of saline–alkali-grown melons were characterized using GC-IMS coupled with VIP analysis. Non-targeted metabolomics using LC-HRMS with alternating ionization modes identified differentially expressed metabolites. Finally, multi-omics correlation analysis integrated nutritional components, volatiles, and characteristic metabolites to establish associative models, revealing intrinsic relationships among quality parameters. This research provides mechanistic insights into varietal differences under contrasting edaphic conditions, offering a scientific basis for optimizing melon cultivation in saline–alkali soils and supporting the sustainable utilization of marginal land resources in China.

## 2. Materials and Methods

### 2.1. Materials and Instruments

As shown in Table 1, Two factors were considered: melon cultivar (‘Yangjiaocui’ and ‘Boyang 9’) and soil type (saline–alkali and non-saline–alkali). Saline–alkali soil ‘Yangjiaocui’ and ‘Boyang 9’ samples were collected in Qing County, Cangzhou City, Hebei Province in April 2025, with the sampled soil exhibiting a water-soluble salt content of 4.8 g/kg and an alkalinity level of 8%. For non-saline–alkali soil samples, the same procedures were followed using samples collected from Weifang City, Shandong Province (soil: 0.7 g/kg salt, 2% alkalinity). For each soil type and each melon variety, 10 uniformly sized, uniformly mature, and mechanically undamaged melon samples were selected as biological replicates. These samples were stored at –1 ± 0.5 °C for subsequent parameter measurements. A portion of the fresh samples was used to determine indicators such as color, texture, moisture content, and soluble solids. The remaining samples were sliced, freeze-dried, ground into powder, sieved through an 80-mesh screen, and stored at –20 °C for future use. Each biological replicate was measured in triplicate (technical replicates).

It should be noted that the saline–alkali soil samples (Hebei Province) and non-saline–alkali soil samples (Shandong Province) were collected from two different geographical locations, which may introduce confounding factors such as climate, baseline soil properties, and local agronomic practices. Therefore, the observed differences between the two cultivation conditions should be interpreted as associative rather than purely causal. Furthermore, detailed records of agronomic practices—including fertilization, irrigation, and pest management—were not available because the samples were collected from local production fields. Consequently, the potential influence of these practices on metabolite profiles cannot be ruled out.

### 2.2. Experimental Methods

#### 2.2.1. Determination of Physical and Chemical Indicators

The weight of each melon variety was measured using an electronic balance (CP114, OHAUS Corporation, Parsippany, NJ, USA). Transverse and longitudinal diameters were determined with a vernier caliper, and the fruit shape index was calculated according to Formula (1). Color parameters (L*, a*, and b*) were measured using a colorimeter (CR-400 Konica Minnolta, Tokyo, Japan). For texture analysis, a standardized sample (1 × 1 × 0.5 cm) was excised from the equatorial region of the fruit. A Texture Profile Analysis (TPA) was then performed using a food physical analyzerr (TMS-PRO FoodTechnology Corporation, Sterling, VA, USA). Key texture parameters, including hardness, cohesiveness, springiness, chewiness, gumminess, and resilience, were derived from the resulting force–time curves using the instrument’s dedicated software.

(1) $$\mathrm{Fruit}\;\mathrm{shape}\;\mathrm{index}=\frac{\mathrm{Fruit}\;\mathrm{length}}{\mathrm{Fruit}\;\mathrm{width}}$$

#### 2.2.2. Determination of Nutrients

Fruits were longitudinally sectioned, and a pulp sample was excised from the equatorial region near the umbilical end (approximately one-third of the fruit length from the stem end) for the determination of moisture content, total soluble solids (TSS), titratable acidity (TA), soluble sugars (SS), and other related indices. Moisture content was analyzed following the national standard method GB/T 5009.3-2003 “Determination of Moisture Content in Food” [9]. TSS was measured with a handheld refractometer (RSD200), TA was determined by sodium hydroxide titration, and SS content was quantified using the anthrone–sulfuric acid method. The sugar–acid ratio and solid–acid ratio, which are commonly adopted for assessing taste balance in fruits, were subsequently calculated according to Equations (2) and (3), respectively [10].

(2) $$\mathrm{Sugar}\text{–}\mathrm{acid}\;\mathrm{ratio}=\frac{\mathrm{Soluble}\;\mathrm{sugars}}{\mathrm{Titratable}\;\mathrm{acids}}$$

(3) $$\mathrm{Solid}\text{–}\mathrm{acid}\;\mathrm{ratio}=\frac{\mathrm{Soluble}\;\mathrm{solids}}{\mathrm{Titratable}\;\mathrm{acids}}\;$$

#### 2.2.3. Determination of Antioxidant Components

Ascorbic acid content was determined according to the Chinese national standard GB 5009.86-2016 “Determination of Vitamin C in Foods” [11]. Total polyphenol and total flavonoid contents were analyzed following the method described by Ali Guler et al. [12]. Briefly, a gallic acid standard (5 mg) was weighed and used to prepare calibration solutions at concentrations of 0, 8, 12, 16, and 40 mg/mL. For each melon sample, 0.2 g of homogenized tissue was weighed into a 10 mL centrifuge tube, mixed with 3.2 mL of 60% ethanol, and subjected to ultrasonic extraction (60 W, 60 min) with thorough shaking. The mixture was centrifuged at 9000× *g* for 5 min, and the supernatant was collected. Subsequently, 10 μL of the extract was combined with 50 μL of Folin–Ciocalteu reagent and allowed to react for 5 min at room temperature in the dark. Then, 750 μL of 7% sodium carbonate solution was added, and the volume was adjusted to 5 mL with distilled water. After incubation in the dark for 120 min, the absorbance of both samples and standards was measured at 760 nm using a microplate reader (Thermo Fisher Scientific, Waltham, MA, USA). Total flavonoid content was determined using rutin as the standard with absorbance measured at 510 nm.

#### 2.2.4. Determination of Mineral Elements

Microwave-assisted digestion was performed by weighing 0.25 g of melon sample into a digestion vessel, adding 6 mL of nitric acid for pre-digestion over 1 h, followed by the addition of 2 mL of hydrogen peroxide. After the digestion process, the resulting solution was diluted to 50 mL with ultrapure water prior to instrumental analysis.

Auto-sampler parameters: sample lift rate: 2 mL/min (0.5 rps) for 40 s; pre-analysis stabilization: 0.4 rps for 30 s; multi-element simultaneous analysis at 0.1 rps.

Quantitative analysis mode: He gas mode, 3 mass units per acquisition point, 3 data acquisition repetitions; integration time: As = 1 s, Se and Cd = 2 s, Pb = 3 s, other elements = 0.3 s.

Specific mass spectrometer operating parameters: RF power: 1600 W, carrier gas flow rate: 1.0 L/min, peristaltic pump speed: 0.1 rps, nebulizer chamber temperature: 2 °C, oxide index: 0.45%, double charge index: 1.01%.

#### 2.2.5. Determination of Volatile Aroma Compounds

Melon aroma components were determined using a Flavor Spec^®^ gas phase ion mobility spectrometer (G.A.S., Dortmund, North Rhine-Westphalia, Germany). For autosampling, 2.0 g of melon sample was placed in a 20 mL headspace vial. The headspace injection conditions were as follows: incubation at 40 °C for 10 min with a shaking speed of 500 r/min, an injection needle temperature of 85 °C, and an injection volume of 1.0 mL [13].

For GC-IMS conditions, the headspace fraction was extracted using a heated injection needle (Agilent Technologies, Santa Clara, CA, USA) and analyzed for volatile organic compounds (VOCs) using 2-methyl-3-heptanone (chromatographically pure) as the internal standard. GC conditions: column temperature 60 °C and maintained constant; operation time of 40 min, and nitrogen (≥99.999%) as the carrier gas. The carrier gas flow rates were 0–2 min: 2 mL/min; 2–10 min: 2–15 mL/min; 10–25 min: 15–100 mL/min; 25–30 min: 100 mL/min.

Method repeatability and calibration: To assess method repeatability, each melon sample was analyzed in triplicate (technical replicates). The relative standard deviations (RSDs) of retention times and peak intensities for major volatile compounds were below 5% and 10%, respectively, indicating satisfactory repeatability. Calibration was performed using the internal standard method (2-methyl-3-heptanone). A series of standard solutions at five concentration levels was prepared to establish calibration curves for target volatiles, with correlation coefficients (R^2^) > 0.99 for all analytes. The limits of detection (LOD) and quantification (LOQ) were calculated as three and ten times the signal-to-noise ratio, respectively.

#### 2.2.6. Qualitative and Quantitative Analysis of Volatile Aroma Compounds

Volatile organic compounds in the samples were collected and analyzed using the VOCal analysis software (Version 4.0.3, G.A.S., Dortmund, North Rhine-Westphalia, Germany) bundled with the instrument. Qualitative analysis of the volatile organic compounds was performed using the NIST and IMS databases integrated within the Library Search software (GCxIMS Library Search V2.2.1). As an internal standard, 2-Methyl-3-heptanone was employed. The content of each volatile aromatic compound was calculated by comparing its peak area with that of the standard compound, as shown in Equation (4):

(4) $$C=\frac{S_{x}\;\times \;C_{0}\;\times \;V\;\times \;1000}{S_{0}\;\times \;m}$$

In the equation: *C* represented the concentration of the analyte, μg/g; *C*_0_ denoted the mass concentration of the internal standard, mg/mL; *S_x_* and *S*_0_ denoted the chromatographic peak areas of the analyte and internal standard, respectively; *m* was the sample mass, g; *V* was the volume of the internal standard, mL; 1000 was the conversion factor.

As this calculation assumes a consistent response factor across all analytes relative to the single internal standard without constructing individual calibration curves, the obtained values are considered semi-quantitative.

#### 2.2.7. Non-Targeted Metabolomics Analysis

Place 100 mg of melon sample into a 2 mL centrifuge tube and add one 6 mm diameter grinding bead. Extract metabolites using 800 μL extraction solution (methanol:water = 4:1 (*v*:*v*)) containing four internal standards (L-2-chlorophenylalanine (0.02 mg/mL), etc.). The mixture was vortexed for 30 s and subjected to ultrasonic extraction at 5 °C and 40 kHz for 30 min. Afterward, the samples were incubated at −20 °C for 30 min and centrifuged at 13,000× *g* for 15 min at 4 °C. The resulting supernatant was carefully transferred and evaporated to dryness under a gentle stream of nitrogen. Transfer the supernatant to an injection vial with an insert tube for instrument analysis. In addition, 20 μL of supernatant was pipetted individually from each sample and then mixed together to serve as a quality control (QC) sample. Each QC sample, matching test samples in volume and processed identically, was inserted every eight test samples during analysis to assess process stability.

Instrument parameters are as follows:

Chromatographic conditions: Sample separation and mass spectrometry detection were performed using two columns (BEHC18 column and BEH Amide column). For the BEHC18 column (100 mm × 2.1 mm i.d., 1.7 μm), mobile phase A was 2% acetonitrile/water (containing 0.1% formic acid), and mobile phase B was acetonitrile (containing 0.1% formic acid). The gradient conditions were: 0–0.5 min held at 2% B, 0.5–7.5 min: linear increase to 35% B, 7.5–13 min: increase to 95% B, 13–14.4 min: hold at 95% B, 14.4–14.5 min: decrease to 2% B, 14.5–16 min: hold at 2% B. Flow rate: 0.40 mL/min. Column temperature: 40 °C.

Mass spectrometry conditions: Sample mass spectrometry signals were acquired using positive and negative ion scanning modes, with a mass range of m/z 70–1050. Ionization voltage: positive ions 3500 V, negative ions −3000 V. Sheath gas 50 psi, auxiliary heating gas 13 psi. Ion source heating temperature 450 °C. Collision energy cycling: 20–40–60 eV. MS1 resolution 70,000, MS2 resolution 17,500.

The LC/MS/MS raw data were preprocessed with Progenesis QI software (Version 3.0, Waters Corporation, Milford, CT, USA). Identification of metabolites was performed by matching MS and MS/MS data against mainstream public databases (such as http://www.hmdb.ca/ (accessed on 11 June 2025); https://metlin.scripps.edu/ (accessed on 12 June 2025), and others) and a self-constructed database supported by Majorbio Biotechnology Co., Ltd. (Shanghai, China) [14,15]. The search parameter was set to signal-to-noise ratio (S/N) ≥ 3, and a result was considered reliable when its S/N exceeded this threshold. Molecular formula prediction was based on the parent ion m/z, possible adduct ions, and isotope peaks from primary mass spectrometry. Metabolites were matched with database entries using a mass deviation threshold of 10 ppm. Daughter ions were matched against the secondary mass spectra of candidate substances, and the weighted mass cosine similarity method was used to select candidate matches. Peak areas served as relative quantitative values for each metabolite. Subsequently, a three-dimensional CSV data matrix (sample information, metabolite name, and MS response intensity) was exported. Metabolic features detected in at least 80% of any sample set were retained [16]. Missing values were imputed with the minimum value in the matrix, and each metabolic signature was normalized to the sum (sum normalization) to minimize errors from sample preparation and instrument instability, yielding a normalized data matrix. Variables from QC samples with a relative standard deviation (RSD) > 30% were excluded, and the remaining data were log10-transformed to generate the final data matrix for subsequent analysis [17].

For metabolite identification within Progenesis QI (Version 3.0, Waters Corporation, Milford, CT, USA), the primary evaluation criteria are mass deviation, isotopic similarity, and secondary fragment similarity. To ensure accuracy, a threshold of 10 ppm for primary mass deviation is set in the scoring criteria for each rating category; matches with a fragment similarity score greater than 30 are considered valid. Based on the scores of these valid matches, the best match is selected as the qualitative result. According to the metabolomics standards initiative (MSI) [18], the identifications reported in this study correspond to Level 2 (putatively annotated compounds), i.e., based on spectral matching against public and in-house databases without confirmation by authentic standards. To provide further granularity, we divided Level 2 into two subcategories: Bi—identification by experimental spectral matching using MS/MS spectra acquired under identical analytical conditions, and Bii—identification by theoretical spectral matching using in silico predicted fragments or literature spectra. The metabolite numbers presented in this work include both Bi and Bii identifications. The retention criteria on the platform were a Fragmentation Score greater than 35 or a Theoretical Fragmentation Score greater than 40.

#### 2.2.8. Integrated Multi-Omics Analysis

Using the DIABLO framework from the “MixOmics” R package, we applied supervised multi-block partial least squares discriminant analysis (sPLS-DA) [19] to identify discriminative features across multiple datasets that account for inter-group variations. To minimize background interference, the variable importance in projection (VIP) method was employed to select features with VIP scores exceeding 1.0 [20], thereby focusing on the most discriminative high-abundance compounds in melon samples. Feature correlations were visualized using the “plotVar” function, while the “cim” function was applied to examine associations between metabolic components and other variables. Correlation heatmaps were generated using the ComplexHeatmap package for intuitive data representation [21]. Additionally, the “cimDiablo” function effectively highlighted multi-omics molecular expression patterns specific to each melon variety.

#### 2.2.9. Statistical Data

Data processing was performed using Microsoft Excel. One-way analysis of variance (ANOVA) followed by Duncan’s multiple range test was conducted with SPSS 23.0, with statistical significance defined at *p* < 0.05. All measurements were carried out in triplicate, and data are presented as mean ± standard deviation. Hierarchical cluster analysis, principal component analysis (PCA), and histogram generation were performed using Origin 2024 and GraphPad Prism 9.5. Further multivariate analysis, including PCA and orthogonal partial least squares-discriminant analysis (OPLS-DA) of volatile compounds, was implemented in SIMCA 14.1. The volatile aroma profiles were visualized using the Reporter plug-in within the Laboratory Analytical Viewer (LAV) software (version 2.2.1, G.A.S., Dortmund, North Rhine-Westphalia, Germany).

## 3. Results

### 3.1. Phenotype, Color, Texture of Melon

**Figure 1:**
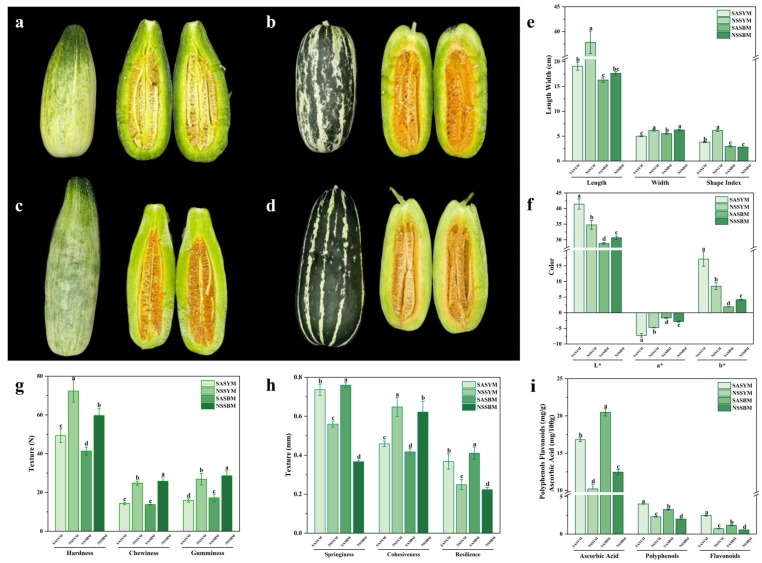
Appearance texture data of four melon varieties. (**a**) SASYM; (**b**) SASBM; (**c**) NSSYM; (**d**) NSSBM; (**e**) Transverse diameter, longitudinal diameter, and shape index of melon; (**f**) Melon color; (**g**) Texture (N); (**h**) Texture (mm). (**i**) Antioxidant composition of four varieties of melon. (Note: Different lowercase letters (a, b, c, d) indicate statistically significant differences between groups (*p* < 0.05); asterisks (*) represent Lab color space indices, including L* (lightness), a* (red-green), and b* (yellow-blue).

The appearance and morphology of melons represent fundamental attributes influencing consumer purchase decisions and overall market acceptability (Figure 1a–d). As illustrated in Figure 1e, the transverse diameter of the melons ranged from 28 ± 1.2 mm to 33 ± 1.8 mm, while the longitudinal diameter varied between 23 ± 1.3 mm and 28 ± 2.6 mm, consistent with an elliptical morphology that is commercially preferred for its handling and visual appeal. Color represents another essential criterion for evaluating fruit quality, significantly affecting consumer perception and market value [22]. The L* value (lightness) ranged from 28.86 to 41.45 (highest in SASYM, lowest in SASBM); a* values ranged from –7.26 to –1.67, indicating a persistent greenish hue typical of unripe or green-fleshed melons; b* values ranged from 1.91 to 17.18 (highest in SASYM, lowest in SASBM), suggesting differential carotenoid accumulation that may reflect variations in ripeness and nutritional quality [23] (Figure 1f).

The textural properties of the melon samples are summarized in Figure 1g,h. Hardness, defined as the force required to achieve a specified deformation and indicative of tissue density and structural integrity [24], ranged from 41.37 ± 2.09 N to 72.34 ± 5.71 N. Resilience, which reflects the degree of shape recovery after force removal and is associated with freshness and ripeness [25], varied between 0.37 ± 0.01 mm and 0.76 ± 0.01 mm. Chewiness, representing the energy needed to masticate a solid sample to a swallowable consistency and reflecting resistance to sustained deformation [26], was recorded in the range of 13.75 ± 0.2 N to 14.35 ± 0.67 N. Adhesiveness, describing the tendency of food to adhere to oral surfaces and influencing texture perception and flavor release, spanned from 15.9 ± 0.92 N to 26.84 ± 2.58 N. Cohesiveness, indicating the strength of internal bonds and the ability of the material to withstand disintegration, ranged from 0.42 ± 0.02 to 0.65 ± 0.05. Overall, melons grown in saline–alkali soils exhibited lower hardness, chewiness, and adhesiveness, but higher resilience, indicating a crisp, elastic texture with reduced resistance to mastication and minimal surface adhesion. In contrast, non-saline–alkali cultivars generally demonstrated higher hardness, chewiness, and adhesiveness, coupled with relatively lower resilience, suggesting a denser, firmer texture with greater oral resistance and slight stickiness.

### 3.2. Analysis of Basic Nutritional Components of Melon

Measured parameters for melons cultivated in saline–alkali and non-saline–alkali soils are summarized in Table 2. Water content, a key factor affecting fruit quality, storability, and transportability [27], ranged from 69.39 ± 1.71% to 89.70 ± 0.09%, with higher values observed in non-saline–alkali samples, indicating a notable environmental influence on moisture retention. Soluble solids content, an important indicator of maturity and flavor closely associated with sugars, acids, and minerals, was higher in saline–alkali cultivated melons, likely reflecting stress-induced osmotic adjustment through sugar accumulation [28], as exemplified by ‘Yangjiaocui’ (11.62 ± 0.08%) and ‘Boyang 9’ (10.63 ± 0.15%). Soluble sugars, which function as energy sources, osmotic regulators, and participants in antioxidant and ripening-related metabolic pathways [29], were also elevated under saline–alkali conditions, with ‘Yangjiaocui’ and ‘Boyang 9’ containing 47.17 ± 0.156% and 44.12 ± 0.418%, respectively, suggesting that such environments may enhance sugar accumulation. Titratable acidity, a critical metric for evaluating sourness and maturation stage, varied between 0.22 ± 0.02% and 0.24 ± 0.01%. The sugar–acid ratio, which significantly influences flavor balance and nutritional synthesis, ranged from 102.76 ± 7.68 to 199.43 ± 6.29. Similarly, the solid–acid ratio, an integrated indicator of sweetness intensity and overall quality, varied from 34.82 ± 1.09 to 49.13 ± 1.38. These findings indicate that saline–alkali cultivation may trigger adaptive stress responses and may enhance certain nutritional parameters. The results suggest that such growing conditions could be associated with increased sweetness and higher nutritional density, but further investigation is needed to establish direct effects on overall fruit quality.

Similar cultivar-dependent variations in antioxidants and fruit quality parameters have been reported in sweet cherry under different ripening phases [30], which is consistent with the possibility that genotype × environment interactions may play a role across different fruit species.

### 3.3. Content of Total Phenolic, Total Flavonoid, and Vitamin C

Vitamin C, an essential nutrient with demonstrated antioxidant properties, contributes to free radical scavenging, oxidative stress mitigation, iron absorption, and immune function enhancement [31]. The Vitamin C content in saline–alkali soil was 1.5 times higher than that in non-saline–alkali soil (*p* < 0.001), with the highest concentration found in SASBM (20.48 mg/100 g).

Polyphenols and flavonoids in melons exhibit diverse biological activities, including antioxidant effects, prevention of chronic diseases such as cardiovascular conditions and cancer, and regulation of metabolic function [32]. As shown in Figure 1i, The SASYM displayed markedly higher contents of these bioactive compounds, with polyphenol and flavonoid levels reaching 4.04 ± 0.03 mg/g and 2.48 ± 0.07 mg/g, respectively, surpassing those of other tested varieties.

### 3.4. Melon Mineral Element Analysis

Melons contain abundant mineral elements; we analyzed 18 elements including K, Na, Ca, Mg, P, B, and Fe. As summarized in Table 3, significant differences in mineral content were observed between the ‘Yangjiaocui’ and ‘Boyang 9’ varieties. K, Ca, Mg, and P were the predominant minerals (concentrations > 100 mg/kg).

Potassium (K) is essential for cellular function and contributes to taste perception by regulating taste cell membrane potential, thereby enhancing sweetness perception [33]. Consistent with this mechanism, salt–alkali-tolerant varieties exhibited higher K content (‘Yangjiaocui’: 25,286.23 mg/kg; ‘Boyang 9’: 27,894.44 mg/kg) and correspondingly higher sweetness. Phosphorus (P) plays a key role in fruit sugar metabolism: it facilitates sugar accumulation, positively correlates with soluble sugar content, and influences fruit firmness and ripening via phosphorus distribution and transport [34]. ‘Boyang 9’ demonstrated significantly higher P content (2043.39 mg/kg in saline–alkali soil; 2224.21 mg/kg in non-saline–alkali soil) compared to ‘Yangjiaocui’. Mg enhances sugar content and fruit quality through its involvement in photosynthesis, carbon–nitrogen metabolism, and sugar accumulation [35], while Mg deficiency adversely affects acidity, firmness, and storage life. Ca contributes to increased fruit firmness, sugar content, and weight, delays softening and senescence, extends shelf life, and improves disease resistance [36]. ‘Yangjiaocui’ exhibited significantly higher Mg (2086.83 mg/kg in saline–alkali soil; 1935.04 mg/kg in non-saline–alkali soil) and Ca (4611.02 mg/kg in saline–alkali soil; 2884.03 mg/kg in non-saline–alkali soil) content than ‘Boyang 9’, which may contribute to its superior sweetness and palatability.

### 3.5. Comprehensive Quality Analysis

#### 3.5.1. Principal Component Analysis

**Figure 2:**
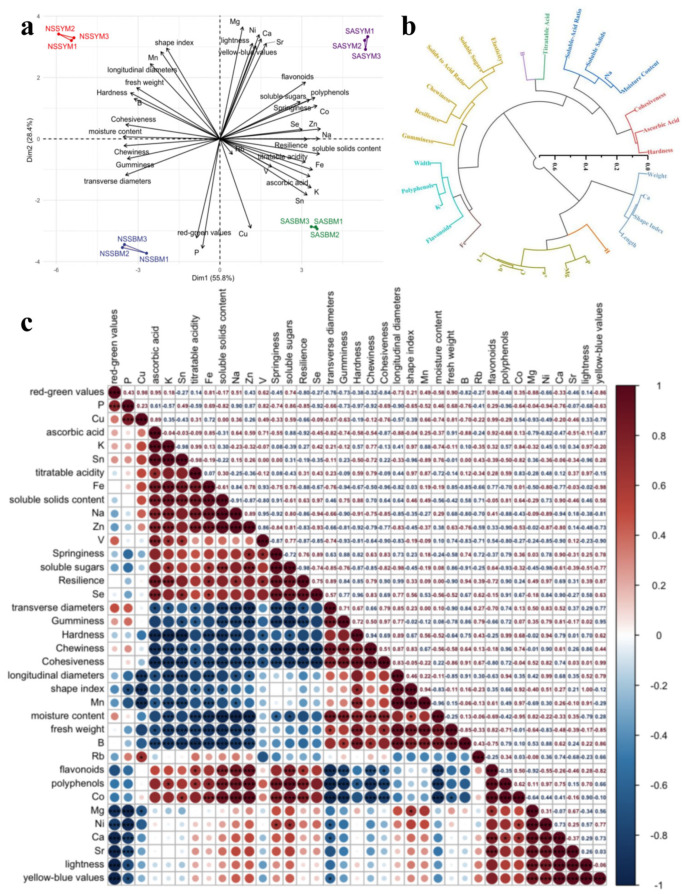
Comprehensive analysis of melon quality. (**a**) Principal Component Analysis Biplot of Fundamental Nutritional Components in Melon. (**b**) Hierarchical cluster analysis of melon indicators. (**c**) Correlation analysis of melon quality indicators. (Circle size indicates correlation magnitude (larger = stronger, smaller = weaker; asterisks indicate significance: * *p* < 0.05, *** *p* < 0.001).

Figure 2a presents a biplot of the principal component analysis, which simultaneously displays the distribution of samples and nutritional components in a reduced two-dimensional space. The first two principal components (PC1 and PC2) collectively explained over half of the total variance, with contribution rates of 55.8% and 28.4%, respectively. Based on the vector lengths and directions in the biplot, PC1 was primarily associated with textural and structural variables (transverse diameter, hardness, and cohesiveness)—the strongest contributors to this component—and clearly separated the saline–alkali soil and non-saline–alkali soil groups along its axis, indicating significant flavor differences among melon varieties under different cultivation conditions. PC2 was mainly characterized by color and morphological parameters (shape index, L*, and b*), which qualitatively exhibited the highest loadings on PC2, further distinguishing ‘Yangjiaocui’ (with higher brightness and yellowness) from ‘Boyang 9’ (displaying darker appearance and lower lightness) along its direction.

#### 3.5.2. Hierarchical Cluster Analysis

Melon quality indicators exhibit significant diversity and complex interrelationships, making direct comprehensive evaluation prone to information redundancy from overlapping metrics. To establish a streamlined assessment framework, hierarchical clustering was applied to generate a dendrogram based on Euclidean distances between indicators, where smaller distances reflect higher similarity. Using a bottom-up agglomerative approach, the algorithm progressively merged the most similar indicators, yielding distinct clusters that provide an intuitive classification of quality parameters.

As illustrated in Figure 2b, the clustering outcome, visualized with distinct colors representing separate categories, classified the 31 indicators into 10 independent clusters. From each cluster, the indicator with the smallest Euclidean distance to the cluster centroid (i.e., the most representative variable) was selected as the core parameter. Using this criterion, hardness, chewiness, fruit weight, hue angle, flavonoids, ascorbic acid, firmness-to-acid ratio, iron, magnesium, and boron were identified as the representative core indicators for their respective clusters. These 10 parameters have been established as the fundamental indicators for evaluating the quality of melons.

#### 3.5.3. Correlation Analysis

Pearson correlation analysis of 37 melon quality indicators (Figure 2c) revealed that hardness exhibited significant positive correlations with chewiness, gumminess, and resilience, suggesting a positive association between firmer tissue structure and mechanical resistance, deformability recovery, and internal cohesion. Conversely, hardness showed significant negative correlations with moisture content, soluble sugars, titratable acidity, and soluble solids content, which is consistent with the characteristic tissue softening and water loss during fruit maturation. Soluble solids content demonstrated a negative correlation with hardness, chewiness, and gumminess, indicating that higher soluble solids were associated with reduced tissue firmness and elasticity. Its positive correlations with soluble sugars, flavonoids, and polyphenols may be related to sugar accumulation and cell wall modification during ripening. Ascorbic acid was positively correlated with both polyphenols and flavonoids; this correlation may reflect their synergistic interaction in antioxidant metabolism, where Vitamin C directly neutralizes free radicals while concurrently promoting phenolic compound biosynthesis, establishing a complementary redox defense system. Significant positive correlations were observed among mineral elements (K, Na, Ca, Mg, P, B, Fe), suggesting coordinated uptake, translocation, and accumulation of these micronutrients. Collectively, mineral elements showed a negative relationship with water content but positive associations with ascorbic acid, polyphenols, and flavonoids, indicating a correlation between mineral enrichment and cellular dehydration as well as enhanced antioxidant metabolism [37]. These interrelationships form an integrated network connecting textural properties, hydration status, carbohydrate metabolism, antioxidant activity, and mineral nutrition.

### 3.6. Analysis of Volatile Compounds

#### 3.6.1. Composition and Content of Volatile Compounds

The sensory quality of melon fruits as perceived by consumers is primarily governed by texture, flavor, and visual appearance, with aroma characteristics being largely determined by volatile compounds [38]. Characteristic food aromas are strongly influenced by volatile flavor compounds and their compositional variations, which play a decisive role in consumer preference [39]. GC-IMS analysis of different melon varieties identified 36 volatile components, comprising 20 esters, five ketones, five alcohols, four acids, and two aldehydes (Table 4), indicating the dominance of esters and ketones in the volatile profile. Significant differences in volatile compound content were observed between ‘Yangjiaocui’ and ‘Boyang 9’, accounting for their distinct aromatic characteristics. M. Wang et al. [40] utilized GC-MS to investigate melon flavor compounds, revealing that during full ripening, ester levels increase substantially under ethylene-mediated gene regulation. Specifically, ethyl acetate, 2-methylpropyl acetate, and 2-methylbutyl acetate were identified as the principal flavor compounds constituting the core aromatic profile, while variations in ethyl acetate, valeraldehyde, and propionaldehyde serve as specific markers for distinguishing flavor differences among melon varieties [41]. Additionally, a recent GC-IMS analysis of 74 melon varieties identified 56 volatile compounds, with esters being the most abundant class, followed by ketones, aldehydes, and alcohols [42]. However, the volatile profiles of the specific cultivars ‘Yangjiaocui’ and ‘Boyang 9’—particularly their comparative characteristics as revealed by GC-IMS—have not been previously detailed. The present study therefore employs GC-IMS to provide a comprehensive characterization of the volatile components of these two cultivars, thereby filling this gap and offering a more nuanced understanding of their distinct aroma traits.

**Figure 3:**
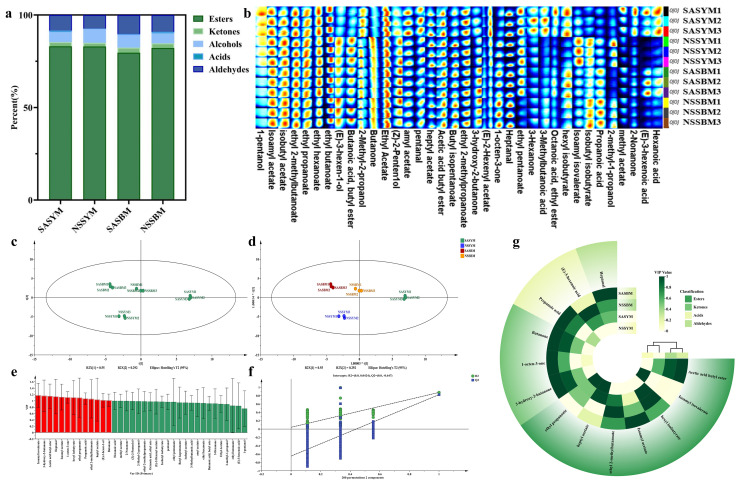
Flavor PCA analysis and cluster heat map. (**a**) Stacked histogram of volatile aroma components composition percentages. (**b**) Flavor fingerprint of melon. (**c**) PCA analysis of volatile aroma components. (**d**) Volatile aroma component OPLS-DA analysis. (**e**) VIP value. (Red represents VIP > 1, green represents VIP < 1). (**f**) Model fit validation. (**g**) Comparative heat map of the content of key volatile aroma components. (Asterisks indicate significance: * *p* < 0.05).

As illustrated in Figure 3a, distinct variations in volatile compound composition were observed among the melon varieties. The saline–alkali cultivated ‘Yangjiaocui’ variety displayed the highest ester content (83.1%), whereas its non-saline–alkali counterpart showed the highest alcohol content (7.3%). The highest proportions of ketones (2.7%) and aldehydes (1.1%) were detected in the saline–alkali grown ‘Boyang 9’, while the non-saline–alkali ‘Boyang 9’ exhibited the greatest abundance of acids (1.1%).

These compositional differences account for the characteristic aroma profiles of each variety. For instance, ethyl valerate contributes sweet, buttery notes reminiscent of apricot and peach with subtle fermented undertones; valeraldehyde provides grassy and apple-like accents; and ethyl acetate delivers aromatic, fruity, and ethereal odors [43]. From a biochemical perspective, these esters are primarily synthesized via alcohol acyltransferase (AAT)-mediated esterification of alcohols and acyl-CoAs, using fatty acids and amino acids as precursors. Further analysis revealed that the characteristic melon-like aroma is primarily attributed to compounds including butyl acetate, 3-hydroxy-2-butanone, and heptanal, which collectively impart sweet, fruity, and fresh herbaceous notes.

#### 3.6.2. Melon Flavor Fingerprinting

The Gallery Plot plugin was utilized to generate and compare fingerprint spectra, effectively highlighting differences in volatile compound composition among the melon samples. Distinctive characteristic fingerprints were established through analysis of specimens representing different varieties and geographical origins. As illustrated in Figure 3b, each horizontal row corresponds to the complete signal profile of an individual sample, while each vertical column represents the signal distribution of a specific volatile aroma component across all analyzed samples.

Volatile compounds including isovalerate esters, ethyl 2-methylbutanoate, and isobutyrate esters were detected at elevated concentrations in both ‘Yangjiaocui’ and ‘Boyang 9’ varieties. These compounds collectively establish an aromatic profile characterized by a sweet fruity foundation, which integrates distinct fruity nuances, such as pineapple, banana, and strawberry, with a mellow sweetness described as both rich and refreshing [44], ultimately defining the characteristic flavor identity of these melon varieties.

Significant differences in volatile organic compound content were observed between saline–alkali and non-saline–alkali soils. Methyl acetate (fruity and floral notes) showed markedly higher concentrations under saline–alkali conditions, as did (E)-3-hexenoic acid (grassy aroma) and 2-nonanone (rose and tea-like notes). These volatiles may function as environmental signaling molecules, and their distribution patterns could potentially influence the sensory properties of the fruits. The results suggest that saline–alkali stress may selectively regulate plant metabolic pathways (e.g., stress-induced shifts in volatile biosynthesis), leading to the specific enrichment of these characteristic aroma compounds. However, further investigation is needed to clarify the exact biological mechanisms underlying these changes.

A principal component analysis model for melon volatiles was established using SIMCA 14.1 software, with 36 aroma components as dependent variables and varieties as independent variables. The first two principal components (PC1 and PC2) collectively explained over half of the variance, with contributions of 55% and 29.2% respectively. ‘Yangjiaocui’ samples clustered primarily in the first and third quadrants, while ‘Boyang 9’ samples were mainly distributed in the second quadrant, demonstrating significant flavor differences between varieties.

OPLS-DA exhibited enhanced discriminatory power, effectively separating melon samples by variety. The model yielded an independent variable fit (R^2^X) of 0.841, dependent variable fit (R^2^Y) of 0.662, and predictive ability (Q^2^) of 0.501. While the Q^2^ value exceeds the commonly accepted threshold of 0.5, it is only marginally above this limit, suggesting that the model’s predictive performance is acceptable but not strong. Furthermore, the moderate discrepancy between R^2^Y (0.662) and Q^2^ (0.501) indicates a potential risk of slight overfitting, although the difference remains within reasonable bounds for metabolomic datasets with limited sample size [45]. Following 200 permutation tests, the regression line’s intersection with the vertical axis below zero (Figure 3f) confirmed the absence of overfitting, validating the model’s reliability for melon variety identification based on aroma profiles.

#### 3.6.3. Screening of Key Aroma Compounds

The contribution of volatile flavor compounds to sample classification was evaluated using variable importance in projection (VIP) analysis derived from partial least squares discriminant modeling. Variables with VIP scores exceeding 1.0 are conventionally considered highly influential in the discrimination model [46], a threshold widely adopted in metabolomics studies for identifying discriminatory markers. As illustrated in Figure 3e, screening of volatile compounds from melon samples grown in saline–alkali soil identified 13 compounds with VIP values above 1.0, common to both ‘Yangjiaocui’ and ‘Boyang 9’ varieties. These included seven esters, three ketones, two acids, and one aldehyde. These significantly influential compounds were: butyl acetate (VIP = 1.14), isopentyl isovalerate (VIP = 1.17), hexyl isobutyrate (VIP = 1.10), isovalerate (VIP = 1.12), ethyl 2-methylbutyrate (VIP = 1.06), ethyl heptanoate (VIP = 1.04), ethyl propionate (VIP = 1.10), 3-hydroxy-2-butanone (VIP = 1.16), 1-nonen-3-one (VIP = 1.11), acetone (VIP = 1.01), propionic acid (VIP = 1.07), (E)-hexenoic acid (VIP = 1.02), and heptanal (VIP = 1.13). From a biological perspective, esters are the predominant contributors to melon aroma, imparting sweet and fruity notes; ketones contribute creamy and fruity nuances; and the selected aldehyde (heptanal) is associated with fresh, green herbaceous characteristics [47]. The predominance of esters among the 13 VIP-selected compounds aligns with the well-established role of ester biosynthesis in determining varietal aroma differences. Thus, the selection of these 13 volatiles not only satisfies the statistical criterion (VIP > 1.0) but also reflects their functional relevance to flavor quality, supporting their use as key markers for variety discrimination under saline–alkali cultivation [48].

Further cluster analysis of the 13 key volatile components revealed that these characteristic compounds effectively differentiated the melon samples, as visualized in the cluster heatmap (Figure 3g). Compounds including heptanal, isoamyl acetate, ethyl 2-methylbutanoate, butyl acetate, and ethyl propanoate were consistently present at relatively high concentrations across all varieties. In contrast, marked varietal differences were observed in the abundance of (E)-3-hexenoic acid, propionic acid, 1-octen-3-one, and hexyl isobutyrate. These findings demonstrate that the integration of flavor fingerprinting with multivariate analysis provides an effective methodological framework for discriminating melon varieties and geographical origins.

### 3.7. Non-Targeted Metabolomics

#### 3.7.1. Overall Metabolome Characteristics

A non-targeted metabolomics approach employing liquid chromatography–high-resolution mass spectrometry in alternating positive and negative ionization modes was utilized to analyze compositional differences in metabolites between saline–alkali and non-saline–alkali soil conditions for both ‘Yangjiaocui’ and ‘Boyang 9’ melon varieties.

**Figure 4:**
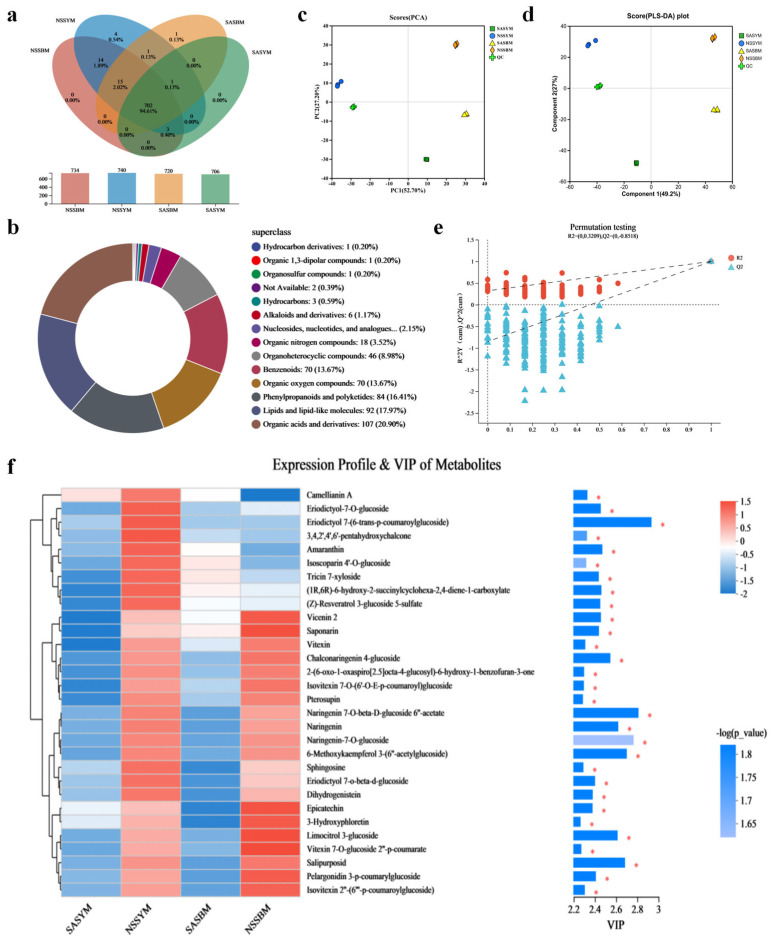
(**a**) Results of Venn analysis. (**b**) Statistical Chart of Compound Classification. (**c**) PCA analysis of metabolic substances in melon. (**d**) PLS-DA analysis. (**e**) Cross-validation diagram of PLS-DA model. (**f**) Analysis of VIP Values. (Asterisks indicate significance: * *p* < 0.05).

As summarized in Figure 4a, a total of 702 distinct compounds were identified across the four sample groups. Under contrasting cultivation conditions, ‘Yangjiaocui’ produced 706 and 740 compounds in saline–alkali and non-saline–alkali soils respectively, while ‘Boyang 9’ yielded 720 and 734 under the same conditions.

Several compounds were consistently detected at elevated levels among all varieties, including 6′′-O-malonylhesperidin, scutellarin, and sisudioside. 6′′-O-Malonylhesperidin has been reported to possess antioxidant and anti-inflammatory activities, and its malonyl moiety may enhance water solubility and potentially contribute to lipid metabolism regulation [49]. Scutellarin is known to exhibit antioxidant and anti-inflammatory functions; it may also enhance sweet taste perception and could support metabolic health through modulation of gut microbiota and improvement of glucose and lipid profiles [50]. While these bioactivities suggest possible links to the observed quality differences among varieties, direct causal relationships remain to be established. Therefore, the functional implications of the differential accumulation of these flavonoids should be interpreted as hypotheses warranting further investigation.

Classification based on structural characteristics was shown in Figure 4b. The primary metabolites in melons mainly include: 107 organic acids and derivatives (20.9%), 92 lipids and lipid-related molecules (17.97%), 84 phenylpropanoid and polyketide compounds (16.41%), aromatic compounds (70 types, 13.65%), organic oxygen compounds (70 types, 13.43%), organic heterocyclic compounds (46 types, 9.59%), organic nitrogen compounds (18 types, 3.84%), nucleotides and derivatives (11 types, 2.15%), and other compounds across 14 categories.

#### 3.7.2. Multivariate Statistical Analysis

Metabolomic profiling revealed distinct differentiation in metabolite distribution patterns between saline–alkali and conventional soil conditions, as well as between the ‘Yangjiaocui’ and ‘Boyang 9’ varieties.

PCA showed a total explained variance of 80.3% (PC1 = 47.70%, PC2 = 32.60%). Model robustness was assessed by cross-validation (Q^2^ = 0.79, based on seven-fold cross-validation). Although the goodness-of-fit R^2^ reached 1.0 (*p* = 0.001), this near-perfect fit should be interpreted with caution, as it may indicate a risk of overfitting given the high dimensionality of the metabolomics data.

PLS-DA further enhanced the separation. The model parameters were as follows: R^2^X = 0.83, R^2^Y = 0.99, Q^2^ = 0.86. A permutation test (*n* = 200) yielded R^2^ intercept = 0.21 and Q^2^ intercept = –0.17, indicating that the original model was not overfitted and had good predictive ability. PC1 primarily distinguished samples by soil type (saline–alkali vs. non-saline–alkali), while PC2 effectively differentiated between the two melon varieties, providing comprehensive insights into the principal factors governing metabolic variation.

Further analysis of intergroup variations in compound content utilizing variable importance in projection (VIP) values is presented in Figure 4f. Under saline–alkali stress conditions, the majority of flavonoid metabolites in melon demonstrated low abundance, whereas these compounds showed elevated expression in non-saline–alkali soil. Key metabolites with VIP values ranging from 2.8 to 2.9 and corresponding −log(p) values approximately between 1.7 and 1.8 included eriodictyol-7-O-glucoside, naringenin-7-O-β-D-glucoside 6′′-acetate, naringenin-7-O-glucoside, 6-methoxykaempferol 3-(6′′-acetylglucoside), and salipurposide. Their marked intergroup differentiation identifies them as potential biomarkers for distinguishing between saline–alkali and non-saline–alkali cultivation environments.

Under both saline–alkali and non-saline–alkali soil conditions, (Z)-resveratrol 3-glucoside 5-sulfate and vicenin-2—both classified as polyphenolic compounds—were consistently detected at elevated expression levels. Glycosylation enhances their water solubility and bioavailability, and subsequent in vivo enzymatic conversion allows transformation into active molecular forms, thereby supporting sustained and regulated biological activity [51]. The stable accumulation of these polyphenols across different cultivation environments suggests that they represent a constitutive metabolic feature of the two melon varieties, rather than a stress-specific response. Given the established roles of polyphenols in antioxidant defense and their potential contribution to flavor perception, their consistent high expression may underpin baseline fruit quality attributes—such as antioxidant capacity and sensory stability—that persist regardless of soil conditions. This finding underscores the importance of variety-specific metabolic traits in determining fruit quality, independent of environmental salinity.

#### 3.7.3. Metabolite Cluster Analysis

**Figure 5:**
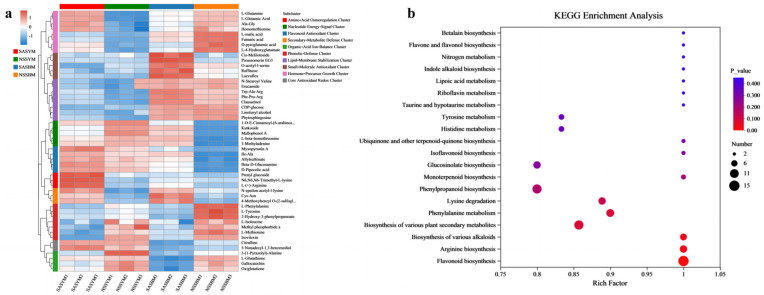
(**a**) Metabolite clustering analysis. (**b**) KEGG pathway enrichment diagram.

Non-targeted metabolomics coupled with hierarchical cluster analysis was employed to systematically characterize metabolic variations between saline–alkali and non-saline–alkali soil conditions, as well as between the ‘Yangjiaocui’ and ‘Boyang 9’ varieties. As demonstrated in Figure 5a, the two varieties exhibit distinctly segregated metabolic profiling patterns.

Significant increases in the levels of multiple amino acids, including L-glutamine, L-glutamic acid, and Ala-Gly, were detected in SASYM. In contrast, NSSYM exhibited marked reductions in organic acids and specific nucleotide metabolites. Concurrently, significant accumulations of isoleucine, α-ketoglutarate, and glutathione were observed in NSSYM [52]. Under saline–alkali soil conditions, ‘Boyang 9’ exhibited significant accumulation of flavonoids and other antioxidant secondary metabolites, including specific flavonoid compounds and quercetin. Furthermore, plants showed concurrent accumulation of organic acids (e.g., azelaic acid and glutaric acid) and additional secondary metabolites. In contrast, ‘Boyang 9’ cultivated in non-saline–alkali soil showed reduced levels of lipid metabolites, including lipid A and phosphatidylcholine, and significant increases in organic acids such as L-malic acid, fumaric acid, and phenylalanine [53].

#### 3.7.4. Pathway Enrichment Analysis

KEGG pathway enrichment analysis (Figure 5b) indicates that flavonoids, as essential plant secondary metabolites, play pivotal roles in antioxidant defense, disease resistance, and signal transduction. This pathway demonstrated an enrichment factor approaching 1, extreme statistical significance, and the highest number of differentially expressed genes, identifying it as the core mechanism underlying the observed metabolic changes. Concurrently, enrichment results encompassed diverse secondary metabolite pathways including alkaloids, terpenoids, and phenolics, suggesting a comprehensive activation of the plant’s defense or adaptive metabolic systems in response to experimental conditions, potentially associated with environmental stress or stage-specific regulation. Tyrosine, as an aromatic amino acid, participates in multiple critical metabolic pathways such as dopamine and phenolic compound biosynthesis, while phenylalanine metabolism serves as the upstream pathway for secondary metabolites including lignin, flavonoids, and phenolic acids. The significant enrichment of both pathways indicates coordinated regulation of upstream metabolism, further explaining the pronounced alterations in downstream products such as flavonoids. The synthesis of various plant secondary metabolites redirects metabolic flux toward flavonoids and other defensive compounds through regulation of aromatic amino acid metabolism, a process that may subsequently influence growth regulation and stress resistance. Although plant hormone signaling pathways showed relatively modest enrichment, differentially expressed hormone-related genes may function as critical regulatory bridges by activating transcription factors that modulate flavonoid biosynthetic genes, thereby orchestrating the integrated response of the aforementioned metabolic network.

### 3.8. Multi-Omics Integration Analysis

#### 3.8.1. Integrative Cross-Communication of Multiple Omics

**Figure 6:**
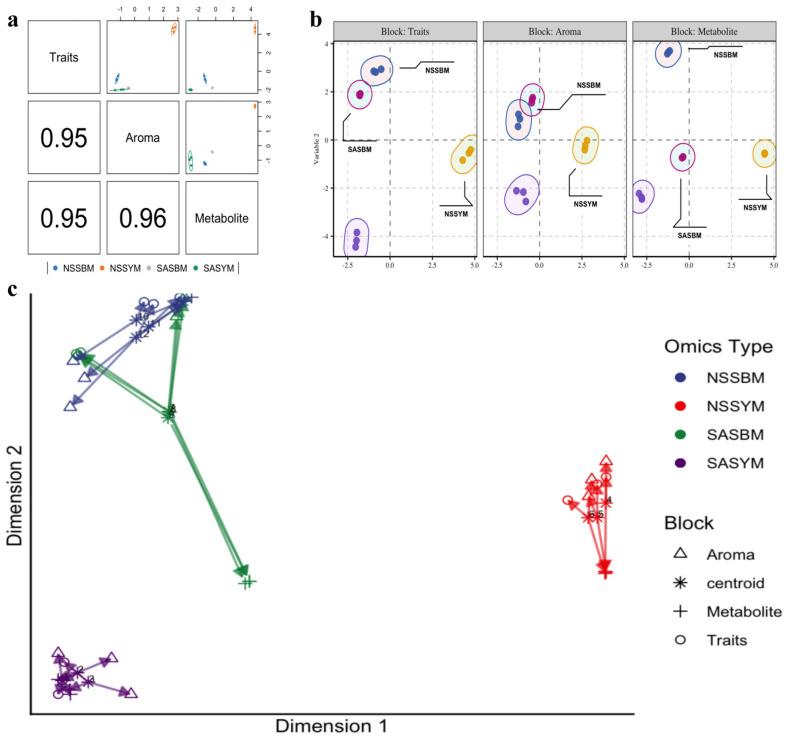
Multi-omics joint analysis. (**a**) Multi-omics DIABLO correlation plot showing Pearson correlations between the first principal component of tri-omics. The correlation among the three datasets supported their integration and the presentation a joint signature; (**b**) Multi-omics joint analysis. Clear discrimination of samples was observed using the signature of basic nutrients, volatile compounds, and characteristics selected by multi-omics DIABLO; (**c**) In the arrow plot, the arrow origin indicates the centroid among all data sets for a given sample, and the tips of the arrows indicate the location of that sample in each block.

Having identified different omics signatures between different samples, our next goal was to explore the interactions of basic nutrients, volatile compounds, and characteristic metabolites, for which we employed DIABLO analysis [54]. This integrated analytical approach effectively captures multi-omics signatures, not only enabling clear discrimination between melon varieties but also elucidating their underlying functional networks (Figure 6a,b).

The arrow plot (Figure 6c) demonstrates that, under different land conditions, ‘Boyang 9’ showed consistent response patterns in both basic nutrients and volatile metabolites, as indicated by analogous distributions of triangular and circular arrows, suggesting that these two substance categories remain stable across varying habitats. In contrast, the cross-arrow distribution representing metabolic pathways exhibited clear separation, reflecting significant remodeling of core metabolic pathways that distinctly differentiate this cultivar under disparate land conditions. Meanwhile, “Yangjiaocui” displayed distinct typological characteristics across multiple omics levels, including basal nutrients, volatile compounds, and signature metabolites.

#### 3.8.2. Multi-Omics Correlation Analysis of Basic Nutrients, Volatile Compounds, and Characteristic Metabolites in Melon

**Figure 7:**
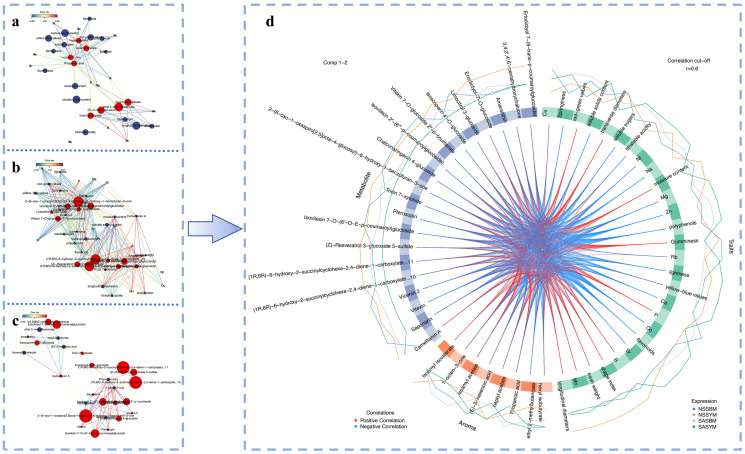
Network analysis of associations among basic nutrients, volatile compounds, and metabolites. (**a**) Association network diagram of basic nutrients and volatile compounds, positive and negative correlations are shown in red and blue; (**b**) Association network diagram of basic nutrients and metabolites, positive and negative correlations are shown in red and blue; (**c**) Association network diagram of volatile compounds and metabolites, positive and negative correlations are shown in red and blue; (**d**) The Circos-plot demonstrated the positive (negative) correlation, denoted as red (blue) lines, between selected multi-omics features. The additional line plots on the outer ring visualize the relative abundance of each feature per experimental group.

Fruit morphological characteristics influence flavor metabolism (Figure 7a), where a shape index correlates with enhanced ester production, likely attributable to increased cell volume upregulating the expression or activity of ester synthases. Color parameters (L*, a*, b*) are linked to specific esters like isoamyl isovalerate, isobutyric acid isobutyl ester, and heptanoic acid heptyl ester, suggesting shared regulatory nodes between pigment metabolism (e.g., carotenoids and chlorophyll) and ester biosynthesis. Soluble sugars and solids positively correlated with multiple esters, providing essential carbon precursors. Titratable acidity correlated with esters such as isovaleric acid isovaleryl ester and hexyl isobutyrate, possibly because an acidic microenvironment favors esterification between organic acids and alcohols. Mineral elements function as critical catalytic cofactors in plant metabolism, directly or indirectly regulating the synthesis of flavor compounds. Specifically, trace elements (e.g., Ni, Mn, Cu) showed strong correlations with short-chain esters, including isobutyric acid isobutyl ester and heptanoic acid ethyl ester, key aroma contributors, potentially by activating acyltransferase-like enzymes. In contrast, alkaline earth metals such as Ca and Sr were associated with propanoic acid, possibly influencing its synthesis and accumulation through calcium-mediated signaling or by maintaining membrane integrity.

Morphological indicators formed a significant positive correlation cluster with flavonoid compounds such as pterostilbene and eriodictyol 7-O-glucoside, suggesting synergistic biosynthesis of multiple flavonoids during fruit development and revealing a close association between fruit morphogenesis and specific flavonoid accumulation. (Figure 7b) In contrast, mineral elements, fresh weight, and color parameters constituted a distinct negative correlation cluster with most flavonoids, reflecting pronounced regulatory relationships among mineral nutrition, biomass accumulation, visual pigmentation, and secondary metabolite biosynthesis. Notably, eriodictyol 7-O-glucoside and soluble solids functioned as central hubs for positive and negative correlations, respectively, implying that their content variations may broadly influence the accumulation patterns of multiple interconnected metabolites.

The metabolic network illustrates co-regulatory relationships between resveratrol glycosides (flavonoid metabolites) and aroma compounds (Figure 7c). Positive correlations (red lines) with volatiles like propionic acid imply co-accumulation, potentially due to shared pathways or flavonoid degradation. Negative correlations (blue lines) among esters, such as ethyl 2-methylbutyrate, indicate competition for biosynthetic precursors. Compounds such as 2-(6-oxo-1-oxaspiro [2.5]oct-4-yl)-6-hydroxy-1-benzofuran-3-one and Eriodictyol 7-(6-trans-p-coumaroylglucoside) are identified as pivotal hub metabolites. These non-volatile compounds structurally integrate the metabolic network by bridging various aromatic compounds and frequently exist in a glycosidic form, serving as stable precursors that liberate volatile aromas upon hydrolysis. Fundamentally, these relationships arise from interconnected metabolism: the phenylpropanoid pathway yields both flavonoids and benzenoid aromatics, while fatty acid metabolism supports ester synthesis and provides flavonoid precursors.

The Circos diagram of multicomponent VIP values (Figure 7d) reveals distinct disparities in nutritional composition and metabolic profiles among samples. SASYM excels in fundamental nutrients, exhibiting the highest concentrations of polyphenols, flavonoids, and ascorbic acid, alongside the most potent antioxidant activity, while its mineral content, particularly macrominerals such as calcium, magnesium, and potassium, also markedly surpasses other groups, further underscoring its superior nutritional value. In terms of aroma, elevated esters like hexyl isobutyrate impart distinct fruity and floral notes; however, this group shows overall reduced metabolite accumulation, likely due to saline–alkaline stress disrupting regular metabolic pathways and limiting end-product synthesis. NSSYM fruits are characterized by prominent shape and vivid color, growing in neutral or mildly alkaline soils. Although their aromatic intensity is relatively modest, metabolic activity remains unimpeded, leading to generally higher levels of diverse metabolites and reflecting robust metabolic vitality. SASYM also demonstrates excellence in basic nutritional metrics (e.g., polyphenols, flavonoids, ascorbic acid), while its volatile profile, featuring compounds such as 1-octen-3-ol, isoamyl acetate, and propanoic acid, imparts a unique blend of mushroom and fruity aromas. However, similar to SASYM, its overall metabolite levels remain comparatively low. In contrast, NSSBM exhibits high accumulation of multiple metabolites (e.g., vitearin, saponarin, vicenin-2) and active metabolic pathway expression.

## 4. Discussion

In this study, we explored how saline–alkali versus non-saline–alkali conditions affect melon quality by integrating physicochemical, volatile, and metabolomic data from two cultivars. Cluster analysis condensed 31 quality indicators into ten representative parameters, among which Vitamin C, flavonoids, solid-to-acid ratio, magnesium, and iron were identified as the most informative for quality assessment.

The volatile profiles were dominated by esters and ketones, a pattern broadly consistent with previous melon studies. However, the relatively high proportion of ketones under saline–alkali conditions suggests that environmental stress may shift the volatile bouquet. Multivariate screening (VIP > 1.0) further defined 13 key aroma compounds that effectively discriminate the two cultivars, supporting the utility of GC-IMS for varietal differentiation. Non-targeted metabolomics revealed 702 metabolites, with marked enrichment of polyphenolic compounds such as (Z)-resveratrol-3-glucoside-5-sulfate and vicenin 2. KEGG pathway analysis identified flavonoid biosynthesis as the most significantly enriched pathway, corroborating observations in other salt-stressed crops and pointing to its central role in antioxidant and defense responses. The concurrent activation of alkaloid and terpenoid pathways indicates a systemic reprogramming of plant secondary metabolism. Finally, DIABLO multi-omics integration uncovered an interactive network linking nutritional components, aroma compounds, and metabolites, providing a systems-level perspective on melon quality regulation.

Several limitations must be acknowledged. First, the saline–alkali and non-saline–alkali soils originated from two different provinces, introducing geographic confounding (climate, baseline soil, agronomic practices); therefore, observed differences are associative rather than causal. Second, only two cultivars and one growing season were studied, limiting generalizability. Third, metabolite identification remained at MSI Level 2. Fourth, sensory evaluation was not performed. Fifth, GC-IMS quantification was semi-quantitative. Sixth, because environmental conditions were not fully crossed with location, a two-way ANOVA was not appropriate; we instead used separate one-way ANOVAs. Future factorial experiments at a single location, with more varieties, multiple seasons, and sensory panels, are needed to validate our conclusions.

## 5. Conclusions

This study systematically compared the physicochemical properties, nutritional components, and volatile aroma compounds (sensory attributes were not formally evaluated) of ‘Yangjiaocui’ and ‘Boyang No. 9’ melons grown under different soil conditions. Cluster analysis identified Vitamin C, flavonoids, solid-to-acid ratio, magnesium, and iron as core indicators for evaluating melon quality. Using GC-IMS technology, 36 volatile compounds were characterized, including 20 esters, five ketones, five alcohols, four acids, and two aldehydes. Among these, butyl acetate, 3-hydroxy-2-butanone, and heptanal contributed to fruity and fresh herbal notes. Multivariate statistical analysis (VIP > 1.0) further screened 13 key aroma compounds, such as isoamyl isovalerate (VIP = 1.17) and butyl acetate (VIP = 1.14). Integration of flavor fingerprint profiles, aroma heatmaps, and OPLS-DA models effectively enabled variety discrimination.

Non-targeted metabolomics identified 702 metabolites, including highly expressed polyphenolic compounds such as (Z)-resveratrol-3-glucoside-5-sulfate and vicenin 2. Glycosylation modifications may enhance their water solubility and bioavailability, potentially facilitating sustained enzymatic conversion and release. These compounds have been reported to possess antioxidant, anti-inflammatory, and other bioactivities, although their specific contributions in this study require further validation.

KEGG pathway enrichment analysis indicated that the flavonoid biosynthesis pathway was significantly enriched, with the highest number of differentially expressed genes, suggesting it as a core responsive pathway that may be linked to antioxidant and defense mechanisms. Concurrent activation of alkaloid and terpenoid pathways pointed to a systemic regulation of plant secondary metabolism.

Building on these findings, DIABLO multi-omics integration revealed an interactive network among nutritional components, aroma constituents, and metabolites. It should be noted that the observed differences between saline–alkali and non-saline–alkali conditions may be influenced by geographic and agronomic confounding factors (e.g., sample origin, farming practices); therefore, the associations described here should be interpreted as correlative rather than directly causal. Nonetheless, this comprehensive analytical approach enhances varietal differentiation and provides a system-level perspective on the factors that may govern melon quality formation.

## Figures and Tables

**Table 1:** Melon variety information.

No.	Name	Variety	Origin
1	saline alkali soil “Yangjiaocui” Melon (SASYM)	“Yangjiaocui”	Saline alkali soil in Qing County, Cangzhou City, Hebei Province.
2	non-saline soil “Yangjiaocui” Melon (NSSYM)	“Yangjiaocui”	Non-saline soil in Weifang City, Shandong Province.
3	saline alkali soil “Boyang 9” Melon (SASBM)	“Boyang 9”	Saline alkali soil in Qing County, Cangzhou City, Hebei Province.
4	non-saline soil “Boyang 9” Melon (NSSBM)	“Boyang 9”	Non-saline soil in Weifang City, Shandong Province.

**Table 2:** Fruit quality indicators in melon.

Varieties	Moisture (%)	Soluble Solids (%)	Soluble Sugars (mg/g)	Titratable Acidity (%)	Sugar–Acid Ratio	Solid–Acid Ratio
SASYM	69.39 ± 1.71 ^d^	11.62 ± 0.08 ^a^	47.17 ± 0.16 ^a^	0.24 ± 0.01 ^a^	199.43 ± 6.29 ^a^	49.13 ± 1.38 ^a^
NSSYM	89.70 ± 0.09 ^a^	6.67 ± 0.18 ^d^	31.03 ± 0.65 ^c^	0.19 ± 0.01 ^b^	161.97 ± 4.81 ^b^	34.82 ± 1.09 ^c^
SASBM	76.42 ± 0.48 ^c^	10.63 ± 0.15 ^b^	44.12 ± 0.42 ^b^	0.23 ± 0.01 ^a^	194.47 ± 6.66 ^a^	46.85 ± 1.67 ^a^
NSSBM	82.05 ± 1.49 ^b^	8.74 ± 0.22 ^c^	22.14 ± 0.61 ^d^	0.22 ± 0.02 ^a^	102.76 ± 7.68 ^c^	40.54 ± 2.72 ^b^

Note: Variety names corresponding to codes are listed in Table 1; different letters within the same column indicate significant differences according to Duncan’s multiple range test (*p* < 0.05).

**Table 3:** Comparative analysis of mineral element content in melons.

Mineral Elements	Concentration (mg/kg)			
	SASYM	NSSYM	SASBM	NSSBM
K	25,286.23 ± 190.43 ^b^	18,187.14 ± 87.15 ^d^	27,894.44 ± 200.63 ^a^	22,191.00 ± 210.39 ^c^
Na	124.45 ± 0.67 ^a^	58.96 ± 2.51 ^d^	102.66 ± 2.30 ^b^	81.26 ± 2.59 ^c^
Ca	4611.02 ± 24.20 ^a^	2884.03 ± 51.41 ^b^	1565.36 ± 10.54 ^c^	1427.97 ± 9.30 ^d^
Mg	2086.83 ± 9.50 ^a^	1935.04 ± 5.76 ^b^	1495.18 ± 12.48 ^c^	1333.72 ± 12.79 ^d^
P	1810.51 ± 29.31 ^c^	1812.18 ± 10.09 ^c^	2043.39 ± 13.44 ^b^	2224.21 ± 18.67 ^a^
B	10.97 ± 0.39 ^b^	15.41 ± 1.21 ^a^	11.36 ± 0.46 ^b^	12.40 ± 0.81 ^b^
Fe	19.71 ± 0.60 ^a^	16.09 ± 0.19 ^c^	19.60 ± 0.99 ^a^	17.95 ± 0.33 ^b^
Sr	26,610.63 ± 345.50 ^a^	13,951.26 ± 41.18 ^b^	6680.56 ± 33.87 ^c^	5991.96 ± 20.19 ^d^
Mn	4078.69 ± 138.11 ^b^	7345.58 ± 82.77 ^a^	3078.03 ± 12.67 ^c^	3084.84 ± 31.35 ^c^
Zn	12,291.31 ± 55.94 ^a^	5620.68 ± 49.75 ^d^	10,030.32 ± 186.59 ^b^	7129.09 ± 70.74 ^c^
Cu	2874.63 ± 26.07 ^b^	2148.82 ± 1.05 ^c^	2965.50 ± 31.06 ^b^	3431.37 ± 133.27 ^a^
Rb	1374.47 ± 9.88 ^b^	1007.74 ± 4.80 ^c^	987.89 ± 7.85 ^d^	1448.58 ± 13.75 ^a^
Sn	754.26 ± 18.58 ^b^	495.19 ± 32.26 ^d^	872.64 ± 17.51 ^a^	664.70 ± 16.16 ^c^
Mo	257.10 ± 8.72 ^c^	177.89 ± 3.50 ^d^	480.04 ± 3.85 ^a^	461.08 ± 6.45 ^b^
Ni	158.21 ± 1.82 ^a^	138.39 ± 8.67 ^b^	113.70 ± 3.65 ^c^	87.66 ± 1.79 ^d^
Se	38.05 ± 0.95 ^a^	30.07 ± 2.67 ^b^	40.13 ± 1.36 ^a^	31.44 ± 1.67 ^b^
Co	30.60 ± 0.74 ^a^	13.42 ± 0.20 ^c^	22.77 ± 0.43 ^b^	13.50 ± 0.54 ^c^
V	15.29 ± 1.23 ^b^	15.15 ± 0.20 ^b^	20.43 ± 1.11 ^a^	13.43 ± 0.43 ^c^

Note: Variety names corresponding to codes are listed in Table 1; different letters within the same column indicate significant differences according to Duncan’s multiple range test (*p* < 0.05).

**Table 4:** Volatile flavor compounds in muskmelon and their contents.

Variety	Name of Aroma Compound	CAS	Molecular Weight	Retention Index	Drift Time	VIP Value	Concentration (μg/g)			
							SASYM	NSSYM	SASBM	NSSBM
Esters	Acetic acid butyl ester	C107879	116.2	814.9	1.61713	1.14766	110.99 ± 0.29 ^b^	107.53 ± 0.57 ^c^	114.42 ± 0.38 ^a^	113.61 ± 1.80 ^a^
	Isoamyl isovalerate	C78933	172.3	1079.9	2.02888	1.17549	47.84 ± 1.37 ^b^	87.68 ± 0.10 ^a^	47.77 ± 0.68 ^b^	47.98 ± 0.31 ^b^
	hexyl isobutyrate	C1629589	172.3	1126	1.46295	1.10407	25.87 ± 0.34 ^a^	13.41 ± 0.67 ^c^	21.12 ± 0.73 ^b^	25.80 ± 0.31 ^a^
	Isoamyl acetate	C96480	130.2	881.4	1.75135	1.11915	127.42 ± 1.26 ^c^	126.03 ± 1.77 ^c^	134.33 ± 0.81 ^a^	130.60 ± 1.21 ^b^
	ethyl 2-methylbutanoate	C1003049	130.2	852.2	1.65358	1.06168	131.88 ± 2.90 ^a^	119.42 ± 0.01 ^b^	127.60 ± 1.39 ^a^	131.87 ± 1.04 ^a^
	heptyl acetate	C40789988	158.2	1110.9	2.03122	1.037	75.99 ± 0.60 ^a^	78.38 ± 0.02 ^a^	64.59 ± 1.26 ^b^	60.92 ± 0.33 ^c^
	ethyl propanoate	C106354	102.1	708.6	1.45922	1.09909	120.08 ± 0.16 ^b^	126.34 ± 0.05 ^a^	114.29 ± 0.15 ^c^	120.12 ± 1.90 ^b^
	Octanoic acid, ethyl ester	C106683	172.3	1196.2	1.4753	0.977417	9.40 ± 0.01 ^a^	5.37 ± 0.09 ^d^	5.95 ± 0.02 ^c^	8.77 ± 0.35 ^b^
	Butyl isopentanoate	C600146	158.2	1045.1	1.38709	0.94597	46.72 ± 3.05 ^b^	49.30 ± 1.00 ^b^	59.04 ± 2.79 ^a^	52.86 ± 4.91 ^b^
	ethyl hexanoate	C110430	144.2	1003.9	1.79043	0.927371	156.48 ± 4.22 ^a^	134.94 ± 2.83 ^c^	114.89 ± 3.83 ^d^	144.49 ± 3.27 ^b^
	Butanoic acid, butyl ester	C120923	144.2	999.1	1.33678	0.917529	1.50 ± 0.05 ^c^	2.50 ± 0.04 ^a^	2.03 ± 0.12 ^b^	2.28 ± 0.01 ^ab^
	amyl acetate	C513860	130.2	916.8	1.76338	0.934788	100.08 ± 1.14 ^a^	75.51 ± 0.41 ^bc^	76.33 ± 0.43 ^b^	74.00 ± 0.14 ^c^
	ethyl pentanoate	C66251	130.2	902.2	1.68366	0.96603	81.54 ± 1.92 ^a^	73.74 ± 0.96 ^b^	37.65 ± 0.98 ^c^	73.20 ± 1.32 ^b^
	ethyl butanoate	C110623	116.2	785.1	1.56108	0.847563	90.24 ± 0.92 ^a^	88.15 ± 0.17 ^b^	80.17 ± 0.26 ^c^	89.55 ± 0.06 ^a^
	isobutyl acetate	C78842	116.2	771.1	1.61651	0.944037	68.46 ± 0.72 ^d^	73.74 ± 0.42 ^b^	77.97 ± 0.38 ^a^	70.82 ± 0.56 ^c^
	ethyl 2-methylpropanoate	C105577	116.2	750.4	1.56287	0.984759	65.66 ± 0.89 ^a^	56.77 ± 1.34 ^b^	53.56 ± 0.74 ^c^	54.60 ± 0.49 ^bc^
	Ethyl Acetate	C123386	88.1	632.7	1.3324	0.902905	303.76 ± 1.03 ^a^	274.72 ± 1.55 ^d^	284.29 ± 0.15 ^b^	277.97 ± 0.75 ^c^
	methyl acetate	C96173	74.1	557.5	1.19916	0.998377	8.81 ± 0.33 ^a^	4.13 ± 0.08 ^d^	6.32 ± 0.35 ^b^	5.83 ± 0.28 ^c^
	Isobutyl isobutyrate	C78853	144.2	922.2	1.31566	0.9725	3.07 ± 0.24 ^d^	5.85 ± 0.28 ^b^	6.38 ± 0.01 ^a^	4.41 ± 0.11 ^c^
	(E)-2-Hexenyl acetate	C590863	142.2	1018.7	1.86149	0.976804	153.41 ± 2.31 ^a^	127.61 ± 1.78 ^b^	133.60 ± 0.31 ^b^	144.37 ± 0.24 ^a^
Ketones	3-hydroxy-2-butanone	C98011	88.1	731.8	1.33931	1.15943	8.53 ± 0.17 ^b^	7.10 ± 0.19 ^c^	9.98 ± 0.06 ^a^	9.69 ± 0.14 ^a^
	1-octen-3-one	C3268493	126.2	954.8	1.26549	1.10752	12.60 ± 1.55 ^c^	13.64 ± 1.70 ^c^	23.07 ± 0.96 ^a^	19.22 ± 1.66 ^b^
	Butanone	C620020	72.1	542.9	1.06563	1.01499	8.53 ± 0.17 ^b^	7.10 ± 0.19 ^c^	9.98 ± 0.06 ^a^	9.69 ± 0.14 ^a^
	2-Nonanone	C107868	142.2	1093.8	1.40458	0.99657	15.52 ± 0.62 ^a^	9.24 ± 0.38 ^c^	9.25 ± 0.38 ^c^	10.14 ± 0.29 ^b^
	3-Hexanone	C928950	100.2	780.4	1.47442	0.915557	2.30 ± 0.13 ^a^	1.51 ± 0.11 ^b^	1.38 ± 0.07 ^bc^	1.28 ± 0.02^c^
Alcohols	(Z)-2-Penten1ol	C763326	86.1	740.4	1.45225	0.992235	6.22 ± 0.15 ^c^	7.59 ± 0.06 ^b^	9.42 ± 0.25 ^a^	9.07 ± 0.33 ^a^
	2-Methyl-2-propanol	C98000	74.1	524.5	1.14467	0.986904	73.77 ± 1.81 ^a^	68.09 ± 1.26 ^b^	61.07 ± 0.46 ^c^	68.99 ± 0.01 ^b^
	2-methyl-1-propanol	C505102	74.1	1128.3	1.16059	0.895976	30.34 ± 0.86 ^c^	63.44 ± 0.52 ^a^	57.57 ± 0.54 ^b^	24.98 ± 0.81 ^d^
	(E)-3-hexen-1-ol	C123513	100.2	858.6	1.24261	0.842387	2.00 ± 0.02 ^d^	3.28 ± 0.10 ^b^	3.52 ± 0.02 ^a^	3.08 ± 0.02 ^c^
	1-pentanol	C107880	88.1	743.7	1.51497	0.757465	2.47 ± 0.14 ^c^	2.91 ± 0.02 ^a^	2.75 ± 0.06 ^b^	2.95 ± 0.05 ^a^
Acids	Propanoic acid	C57556	74.1	732.7	1.09903	1.07201	1.23 ± 0.01 ^d^	1.7 ± 0.04 ^c^	2.55 ± 0.03 ^a^	2.27 ± 0.02 ^b^
	(E)-3-hexenoic acid	C116096	114.1	992.1	1.5898	1.02103	5.87 ± 0.26 ^b^	1.45 ± 0.04 ^d^	2.54 ± 0.09 ^c^	7.66 ± 0.09 ^a^
	Hexanoic acid	C589822	116.2	991.1	1.65646	0.999163	4.44 ± 0.12 ^a^	1.75 ± 0.11 ^d^	2.20 ± 0.01 ^c^	3.91 ± 0.05 ^b^
	3-Methylbutanoic acid	C111273	102.1	860.1	1.49374	0.937729	3.28 ± 0.01 ^a^	1.39 ± 0.01 ^d^	2.12 ± 0.07 ^b^	1.79 ± 0.07 ^c^
Aldehydes	Heptanal	C106707	114.2	939.4	1.69925	1.13491	153.28 ± 0.66 ^b^	127.99 ± 11.33 ^c^	185.19 ± 10.41 ^a^	163.71 ± 2.59 ^b^
	pentanal	C539822	86.1	720.2	1.41772	0.966564	22.38 ± 0.31 ^a^	16.19 ± 0.24 ^d^	18.00 ± 0.05 ^b^	17.30 ± 0.14 ^c^

Note: Variety names corresponding to codes are listed in Table 1; different letters within the same column indicate significant differences according to Duncan’s multiple range test (*p* < 0.05).

## Data Availability

The original contributions presented in the study are included in the article, further inquiries can be directed to the corresponding author.
